# Manpixiao Decoction Halted the Malignant Transformation of Precancerous Lesions of Gastric Cancer: From Network Prediction to *In-Vivo* Verification

**DOI:** 10.3389/fphar.2022.927731

**Published:** 2022-08-05

**Authors:** Yuan Li, Tao Li, Jiena Chen, Haocheng Zheng, Yicong Li, Fuhao Chu, Sici Wang, Ping Li, Jie Lin, Zeqi Su, Xia Ding

**Affiliations:** ^1^ National Institute of Traditional Chinese Medicine Constitution and Preventive Treatment of Diseases, Beijing University of Chinese Medicine, Beijing, China; ^2^ School of Traditional Chinese Medicine, Beijing University of Chinese Medicine, Beijing, China; ^3^ Research Center for Spleen and Stomach Diseases of Traditional Chinese Medicine, Beijing University of Chinese Medicine, Beijing, China; ^4^ Dongzhimen Hospital, Beijing University of Chinese Medicine, Beijing, China; ^5^ Oncology Department, Dongfang Hospital, Beijing University of Chinese Medicine, Beijing, China; ^6^ Institute of Regulatory Science for Traditional Chinese Medicine, Beijing University of Chinese Medicine, Beijing, China; ^7^ Beijing Research Institute of Chinese Medicine, Beijing University of Chinese Medicine, Beijing, China

**Keywords:** Carcinogenesis, dysplasia, Manpixiao, traditional Chinese medicine, PI3K-AKT, epithelial-mesenchymal transition (EMT)

## Abstract

Manpixiao decoction (MPX), a traditional Chinese medicine formula, is mainly used to improve the gastric mucosal pathology and stomach discomfort in patients with gastric precancerous lesions. Precancerous lesion of gastric cancer (PLGC) refers to intestinal metaplasia and/or dysplasia based on gastric mucosal atrophy. Effective prevention and treatment of PLGC is of great significance to reduce the incidence of gastric cancer. Because of the complexity of the etiology and pathogenesis of PLGC, there is no unified and effective treatment plan in western medicine. In recent years, traditional Chinese medicine has shown obvious advantages in the treatment of PLGC and the prevention of its further progression to gastric cancer, relying on its multi-approach and multi-target comprehensive intervention characteristics. This study is designed to examine the protective effect of MPX against PLGC and further to reveal the engaged mechanism via integrating network pharmacology and *in vivo* experimental evidence. Network pharmacology results demonstrated that inflammation, immune responses, and angiogenesis might be associated with the efficacy of MPX in the treatment of PLGC, in which the PI3K-Akt, cellular senescence, P53 and protein processing in endoplasmic reticulum were involved. Then, we established a rat model of PLGC using a combination of N-methyl-N′-nitro-N-nitrosoguanidine (MNNG), sodium salicylate, irregular fasting, and ranitidine, and observed the effects after MPX treatment. Our result showed that MPX improved the pathological condition of gastric mucosa in PLGC rats and reduced the incidence of gastric cancer. Next, the analysis of serum inflammatory cytokines showed that MPX reduced the inflammation-related cytokines (such as IL-1α, IL-7, CSF-1, and CSF-3) in the serum. Additionally, MPX also had a regulation effect on the “protein/protein phosphorylation-signaling pathway” network in the core region of the PLGC rats. It is showed that MPX can inhibit the phosphorylation of PI3K-AKT, and downregulates the EGFR, β-catenin, and N-cadherin protein levels. These results indicate that MPX halted the PLGC progression through inhibiting EGFR-PI3K-AKT related epithelial-mesenchymal transition process.

## Introduction

Gastric cancer (GC) is the fifth most common type of cancers and the fourth leading cause of cancer related death (Sung et al., 2021). Its occurrence often goes through a Correa’s cascade and precancerous lesion of gastric cancer (PLGC) including gastric metaplasia and dysplasia are essential stages in the multistage progression from normal gastric mucosa to gastric adenocarcinoma (Correa et al., 1975). Among the risk factors leading to the premalignant lesions of gastric mucosa, long standing chronic inflammation induced by *Helicobacter pylori* (*H. pylori*) infection, genetic susceptibility, diet, and smoking play pivotal roles in driving malignant transformation of mucus (Thapa et al., 2019; El Khadir et al., 2020; Eusebi et al., 2020). Currently, the major approach to reduce the mortality rate of GC are early diagnosis and intervention for PLGC. Early diagnosis relies mostly on gastroscopy screening, which is cost-effective in moderate to high-risk populations, meanwhile, other highly sensitive methods are increasingly being used to detect the early GC (Amal et al., 2016; Zagari et al., 2017; Fan et al., 2021; Huang et al., 2022). Unfortunately, the main contribution of drugs such as COX-1/2 inhibitors and nonsteroidal anti-inflammatory drugs are to improve symptoms in patients, there is still a lack of medicine to reverse or improve the histopathology in patients with PLGC (Pimentel-Nunes et al., 2019). Therefore, seeking an effective medicine that can halt the cancerous transformation of PLGC and even reverse PLGC is urgently needed.

Malignant transformation due to chronic inflammatory responses in the gastric mucosa is a process of multi-factor participation, multi-cellular regulation and multi-stage evolution. More than 90% of gastric adenocarcinoma is the final outcome caused by chronic inflammation and *H. pylori* infection is related to the majority of gastric cancer (Bockerstett and DiPaolo, 2017). Eradication of *H. pylori* or anti-inflammatory therapy exclusively on the development of premalignant conditions unable to decrease the cancer risk substantially, implying a driving role of crosstalk between the epithelial cells and environmental components in cancer development (Wong et al., 2004; Leung et al., 2006). Chronic inflammation induced by infections could disrupt the homeostasis of gastric environment, lead to gastric mucosa atrophy and gastric acid secretion disorder, and establish a pro-cancer environment to further promote gastric mucosa differentiation with characteristics of tumor cells like epithelial-mesenchymal transition (EMT) (Ma et al., 2016; Courtois et al., 2021). Bioinformatics analysis, as an important analysis method of systematic biology, provide us a new way to explore the core molecular events behind the GC evolution comprehensively (Wörheide et al., 2021).

Traditional Chinese medicine (TCM), as a novel therapeutic strategy under guidance of TCM theory, exhibited multi-target and multi-pathway protective effects on gastric mucosa via resisting oxidation, affecting cell proliferation and differentiation, and regulating immunity (Xu et al., 2022). Many clinical trials and preclinical studies revealed the potential therapeutic effects of TCM prescription on PLGC (Tang et al., 2016; Xu et al., 2018; Wang et al., 2020). Manpixiao decoction (MPX), a TCM formula, is mainly used to improve histopathological changes of the gastric mucosa and alleviate discomfort symptoms in patients with PLGC. However, the molecular mechanism behind the role of MPX in PLGC treatment is not clear. Network pharmacology provide a powerful tool for us to analyze the multi-target and multi-pathway regulating effects through constructing a component-target-pathway relationship network based on the systems biology and multi-directional pharmacology.

In this study, we apply bioinformatics analysis methods to discover the key molecular groups and signaling pathway networks that affecting the process of chronic gastritis malignant transformation, and explore the molecular mechanism of multi-stage pathology evolution. Then, focusing on the critical phases and molecular events, we explored the possible anti-PLGC mechanisms of MPX by integrating pharmacochemistry and network pharmacological analysis. Furthermore, a rat model of PLGC using a combination of N-methyl-N′-nitro-N-nitrosoguanidine (MNNG), sodium salicylate, irregular fasting, and ranitidine was established and the hematoxylin-eosin staining, Luminex multiplex assay, protein microarray detection as well as western blot were used to validate its molecular mechanism in treatment of PLGC.

## Materials and Methods

### LC-MS/MS Analysis of the Serum Containing MPX

The serum containing MPX was collected from Wistar male rats, which were intragastric administrated with MPX continuously for 8 weeks. One hour after the last dose, whole blood was collected from the rat and left for 30 min, centrifuged at 1,500 g for 10 min, and the upper serum was obtained. Then, 300 μL of serum sample was transferred into a 1.5 ml centrifuge tube, add 900 μL acetonitrile, vortex mixing for 3 min and centrifuged for 10 min under 13,000 g at 4°C. The supernatant was taken and dried under nitrogen at 40°C. The obtained residue was added with 300 μL methanol. After ultrasonic suspension, centrifuged at 13,000 g for 10 min, the supernatant was finally filtered with a microporous membrane of 0.22 μm pore size and stored at 4°C before use. In addition, quality control (QC) samples were prepared by mixing an equal amount of each MPX sample (20 μL) and were used to evaluate the stability of the system. The QC samples were repetitively analyzed after every three tested samples.

Ultra-performance liquid chromatography in tandem with mass spectrometry (UPLC-Q-Exactive) (Thermo Fisher Scientific) analysis was used to assess the main ingredients of MPX. UHPLC was conducted in tandem with mass spectrometry using a Thermo fisher U3000 UHPLC and Thermo Scientific Q Exactive mass spectrometer with an ESI source and the following parameters: mobile phase (A) 0.1% Formic Acid (v/v) in Acetonitrile, and (B) 0.1% Formic Acid in (v/v) Water; injection volume 5 μL; column temperature 35°C, using a gradient elution mode. The UHPLC system consisted of an Acquity UPLC BEH C18 (1.7 µm, 3 mm × 150 mm, 1/pkg) (waters, United States) with a 0.3 ml/min flow rate.

### Identification of Key Genes Associated With PLGC

Microarray-based data about expressions of different stages of PLGC or early gastric adenocarcinoma (EGC), containing two Gene Expression Omnibus (GEO) datasets, GSE55696 (Xu et al., 2014) and GSE130823 (Zhang et al., 2020), were obtained from official website of GEO (https://www.ncbi.nlm.nih.gov/geo/) *via* GEOquery R package. The annotaion function in Sangerbox (http://soft.sangerbox.com/) software was used to annotate the probe. GSE55696 includes 19 low-grade intraepithelial neoplasia (LGIN), 20 high-grade intraepithelial neoplasia (HGIN), 19 EGC, and 19 chronic gastritis (CG) samples. GSE130823 includes 17 LGIN, 14 HGIN, 16 EGC, and 47 paired inflammation controls. As stomach adenocarcinoma (STAD) is the most common form of GC and regarded as the final stage of the Correa cascade (Correa and Piazuelo, 2012; Bockerstett and DiPaolo, 2017), RNA sequencing data of STAD samples with clinical information in TCGA were adopted and downloaded from the UCSC Xena browser (Goldman et al., 2020) (https://xenabrowser.net/). After removing samples with 0-day follow-up duration and incomplete clinical information, 343 STAD samples and 30 adjacent samples were obtained. The above two data series were integrated into one GEO dataset (*n* = 171) *via* R package “sva” for removing batch effects and other unwanted variation in two datasets (Leek et al., 2014). The average RNA expression values were taken when duplicate data were found, the highest mean values were reversed. Following the correction of background effect, quantile normalization and log2 transformation, the datasets were used for subsequent analyses. A false discovery rate (FDR) adjusted *p*-value < 0.05 and an absolute value of log2 (fold change) > 1 were considered as the criteria for differential expressed genes (DEGs) identification. Volcanos were conducted by using R package “ggscatter” (Wodrich et al., 2021). To further understand the potential mechanisms of the signature of different stage of PLGC, Gene Set Enrichment Analysis (GSEA), pathway enrichment analysis were performed (Rehman et al., 2021).

### Network Pharmacology Prediction of MPX in the Treatment of PLGC

The molecules obtained by LC-MS/MS were standardized using PubChem (Kim et al., 2019) database. Targets were predicted with the SMILES (Simplified Molecular Input Line Entry System) of molecules by Swiss (Guex and Peitsch, 1997), and only targets with a predicted score greater than 0 were included. The Search Tool for the Retrieval of Interacting Genes/Proteins (STRING) platform (Szklarczyk et al., 2015) was utilized to get correlative targets of MPX and PLGC separately. The intersection of drug targets and disease targets were considered potential therapeutic targets.

The common targets were used to construct the Protein-Protein interaction (PPI) network with the minimum required interaction score of 0.400. Subsequently, the topological property of the PPI network was analyzed using Cytoscape 3.9.1 (Shannon et al., 2003). The modules were extracted by MCODE (Bader and Hogue, 2003) using a node score cutoff of 0.2 and K-core of 2. To analyze the enrichment and function of genes with the gene ontology (GO) functional annotations and Kyoto Encyclopedia of Genes and Genomes (KEGG) pathway, org. Hs.eg.db (Carlson et al., 2019) and clusterProfiler (Yu et al., 2012) packages in R Studio (R Core Team, 2013) were used.

### Animals and Medicine

SPF Wistar male rats (3–4 weeks of age), weighing between 80 and 100 g were purchased from the Sibef Biotechnology Co., LTD. (Beijing), and raised under specific pathogen-free (SPF) conditions. The experimental protocols were approved by the Animal Research Ethics Committee of Beijing University of Chinese Medicine. MNNG was purchased from the TCI (Shanghai) Huacheng Industrial Development Corporation Ltd. (Shanghai, China). Sodium salicylate was purchased from the Sinopharm Chemical Reagent Corporation Ltd. (Beijing, China). Granulated SPF-grade rat fodder containing 0.05% ranitidine was purchased from the Beijing Keao Xieli Feed Corporation Ltd.

MPX was purchased from the granule pharmacy of Dongzhimen Hospital, Beijing University of Chinese Medicine, 17.15 g granule per packet (equivalent to 145 g herbal medicine). The adult body weight was calculated as 70 kg, and the daily dose of granules was 1.5435 g/100 g (equivalent to 13.05 g/100 g of decoction pieces) according to the conversion coefficient of the body surface area between rats and adults by 6.3 times. This experiment was conducted in Research Center for Spleen and Stomach Diseases of Traditional Chinese Medicine, Beijing University of Chinese Medicine.

### Establishment of PLGC Rat Model and Pharmacological Treatment

Animals received food and water under the SPF conditions with a standard 12 h light/dark cycle, a temperature of 22 ± 2°C, and relative humidity of 60 ± 5%. After one week of acclimatization, all the 62 rats were randomly divided into the control group (*n* = 24) and model group (*n* = 38). The rats of the control group were given normal chow and water. The rats of the model group were given multiple factors stimulation, including free to drink 120 μg/ml of MNNG aqueous solution and feed with granular SPF-grade fodder containing 0.05% ranitidine. Besides, on every Tuesday and Friday, abrosia and gavage of 0.05 ml/kg 2% sodium salicylate solution were given to rats of the model group. Normal feeding and gavage of 0.05 ml/kg water were given to rats of the control group.

Based on our previous experiments, the PLGC model was established on the 32nd week (Yu et al., 2020; Chu et al., 2021), and 10 rats from each group were sacrificed for model evaluation. Then, the remaining 28 rats of model group were randomly divided into model group and MPX group. During the period from the 32nd week to the 40th week, two groups of rats received both MNNG multiple factors-induced and pharmacological treatment. MPX at 1 ml/100 g was intragastrically administered to the rats in the MPX group once a day, while rats in control group and model group received water (1 ml/100 g) once daily. The flow chart of the experiment is shown in Supplementary Material S1. At the end of the 40th week, all animals were anesthetized by intraperitoneal injection with 0.2 ml/100 g 2% sodium pentobarbital after 12 h fasting, and the whole stomach was harvested immediately.

### Histological and Immunohistochemistry Evaluation

For histopathological analysis, stomach tissues were fixed in 10% neutral formalin overnight. Sections of the gastric glands were sliced at a thickness of 5 μm and stained with hematoxylin and eosin (H&E) to score the degree of gastritis and gastric carcinoma according to the Updated Sydney System and Japanese classification of gastric carcinoma (Manfred and Alexander, 2001; Japanese Gastric Cancer Association., 2011). Any damage or change in morphology of gastric tissue were examined through microscope. After deparaffinization and rehydration for the remaining sections, heat-induced antigen retrieval was performed by heating the slides for 17 min at 95°C in sodium citrate buffer (pH 6.0). Endogenous peroxidase activity was then blocked by incubating slides in 3% hydrogen peroxide/ethanol for an additional 10 min before incubating at room temperature for 2 h with a 1:1,000 dilution of rabbit anti-PCNA (#10205-2-AP, Proteintech). Bound antibodies were dected by using a conventional streptavidin-biotin method according to the manufacturer’s instrutions (#PV-9001, ZSGB-BIO). The reaction was visualized by 3,3′-diaminobenzidine (DAB) using the DAB Substrate Kit (#ZLI-9018, ZSGB-BIO) and slides were then counterstained with hematoxylin (#C0107, Beyotime).

### Luminex Liquid Suspension Chip Assay

The blood serum was collected for detecting the expression levels of inflammation-related cytokines. Luminex liquid suspension chip conjugated highly specific captured monoclonal antibodies to polystyrene microspheres labeled with different fluorescence. When these precoated microspheres were premixed and incubated with the blood samples, the target was captured by the microspheres and biotin-labeled phyalginin-labeled streptavidin (SA-PE) was added for signal amplification to achieve effective detection of a variety of trace cytokines. The assay was conducted according to manufacturer’s protocol by using Bio-Plex Pro Rat 23-plex Cytokine Grp I Panel (#12005641) with Bio-Plex Magpix System (Bio-rad, United States) in Wayen Biotechnologies Shanghai, Inc.

### Protein Microarray Detection

The gastric tissues of rats were cut and separated from the pylorus and cardia. Under low temperature, the gastric tissues were cut along the greater curvature of the stomach, washed quickly with cold saline, and dried with filter paper. The gastric mucosa tissue (not muscle layer) was scraped with a weighing spoon and placed in a cryopreservation tube. The tissue was quickly put into liquid nitrogen for freezing and then transferred to a refrigerator at -80°C for preservation. CSP100 Plus chip (Full Moon, United States) was used to analyze the key proteins and protein phosphorylation levels involved in 16 tumor-related signal pathways according to manufacturer’s protocol in Wayen Biotechnologies Shanghai, Inc.

### Western Blot Assay

The gastric mucosal tissues of rats were weighed, and then placed into the RIPA lysis buffer (BN25012, Bairuiji, China) containing cell lysis magnetic beads prepared in advance. The samples were ground with a tissue crushing homogenator and the supernatant was retained for BCA assay and further preparation for SDS-PAGE. The separated bands were transferred onto nitrocellulose membrane (Merck Millipore, United States). The membrane was blocked with 5% skim milk at room temperature for 30 min and then incubated with primary antibodies overnight at 4°C. The primary antibodies including: Anti-EGFR antibody (#4267, CST), Anti-PI3 Kinase antibody (#67071-1-Ig, Proteintech), Anti-p-AKT antibody (#4060, CST), Anti-AKT antibody (#4691, CST), Anti-N-cadherin antibody (#14215S, CST), Anti-E-cadherin antibody (#60335-1-Ig, Proteintech) Anti-β-Catenin antibody (#610154, CST), Anti-Vimentin antibody (#60330-1-Ig, Proteintech), Anti-GAPDH antibody (#5174, CST). After secondary antibody incubation, the membranes were washed 3 times and detected by West Pico PLUS Chemiluminescence substrate (#34577, Thermo, United States) and images were obtained using the ChemiScope 3,000 detection system (Qingxiang, Shanghai, China).

## Results

### Characterization of Chemical Constituents in MPX

**FIGURE 1:**
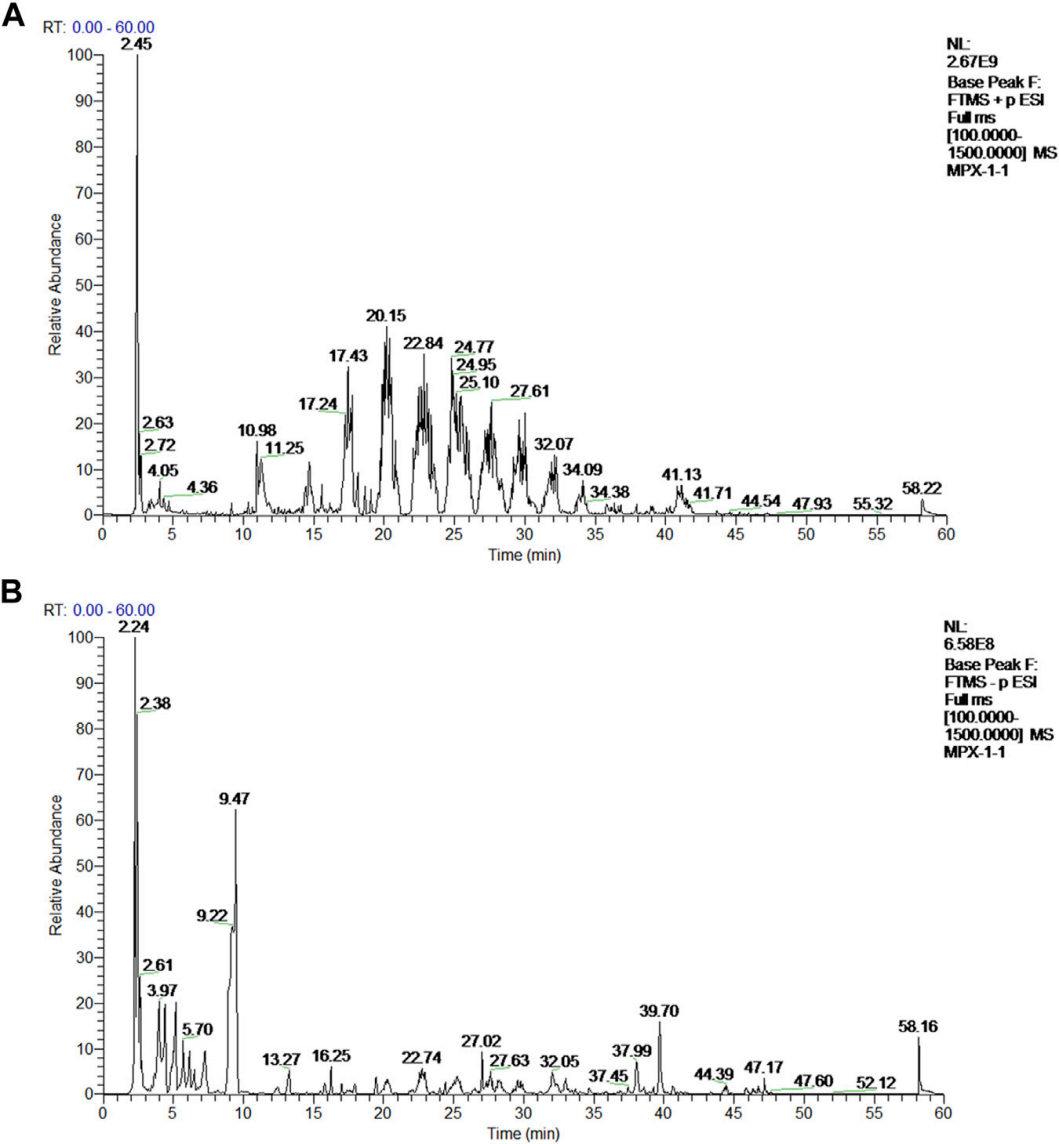
Mass spectrum chromatograms of MPX. **(A)** Positive ion mode base peak chromatogram (BPI) of rat serum samples after MPX intervention; **(B)** Negative ion mode base peak chromatogram (BPI) of rat serum samples after MPX intervention.

The active ingredients in rat serum samples after MPX intervention were detected by UPLC-Q-Exactive-MS. The representative typical base peak chromatograms (BPCs) of drug-contained serum were shown in Figure 1. Compared with the negative ion mode BPCs, positive ion mode BPCs showed more observable peaks in all samples. Based on the OTCML database, 426 and 459 compounds in the positive and negative ion modes of the mass spectra were tentatively identified. Furthermore, through the previous reference literatures, we confirmed 46 main active principles in MPX-containing serum, which include 3 phenolic acids, 4 tanshinones, 9 alkaloids, 19 flavonoids, 5 terpenoids and 6 other components (Supplementary Material S2).

### Characterization of the Potential Targets in the Progress of Carcinogenesis

**FIGURE 2:**
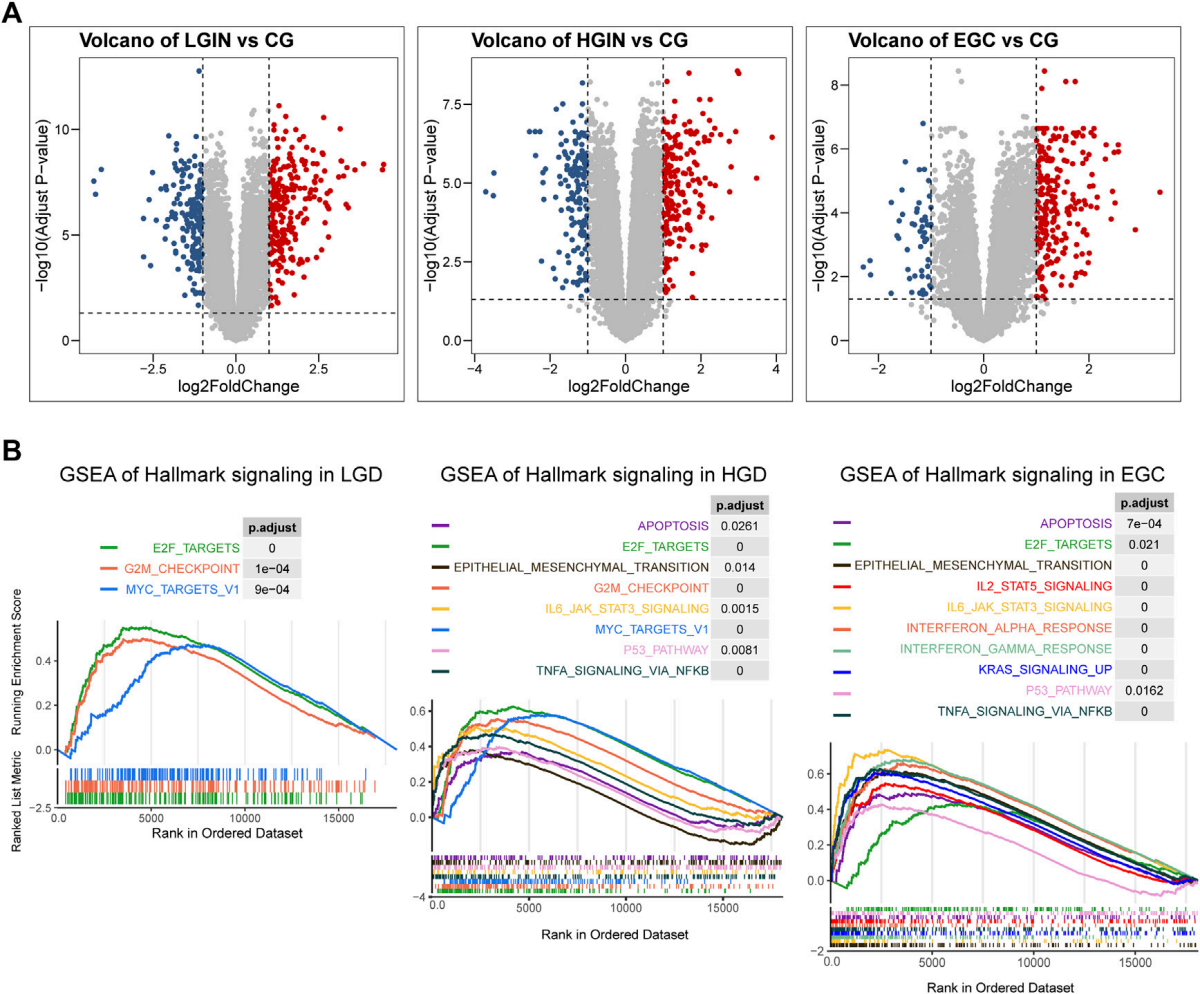
Differential expressed genes (DEGs) and the result of gene set enrichment analysis (GSEA) in PLGC progression to EGC. **(A)** Volcano plots of DEGs in LGIN and CG, HIGH and CG, EGC and CG; **(B)** GSEA of hallmark gene sets in LGIN, HGIN and EGC. LGIN: low-grade intraepithelial neoplasia, HGIN: high-grade intraepithelial neoplasia, EGC: early gastric adenocarcinoma, CG: chronic gastritis.

Based on the cut-off criteria of |logFC| > 1.0 and adj. *p* < 0.05, a total of 417 DEGs were found in LGIN and CG, 367 DEGs in HGIN and CG, 292 DEGs in EGC and CG (Figure 2A, Supplementary Material S3). The degree of activation in patients’ immune- and malignancy-related pathways is related to their prognosis of PLGC. Therefore, we used GSEA to evaluate the activity of pathways in PLGC patients. We found that the activity of immune and malignancy-related pathways gradually increased with pathological progression (Figure 2B). This activity of immune-related pathways was inclusive of several processes: IL6/JAK/STAT3 pathway, TNFA signaling *via* NFKB, IL2/STAT5 pathway, Interferon alpha response and Interferon gamma response. Besides, malignant-related pathways were significantly activated with the progressive of PLGC, such as EMT, P53 pathway and KRAS signaling.

### Potential Targets and Mechanisms of MPX Halted the Malignant Transformation of PLGC

**FIGURE 3:**
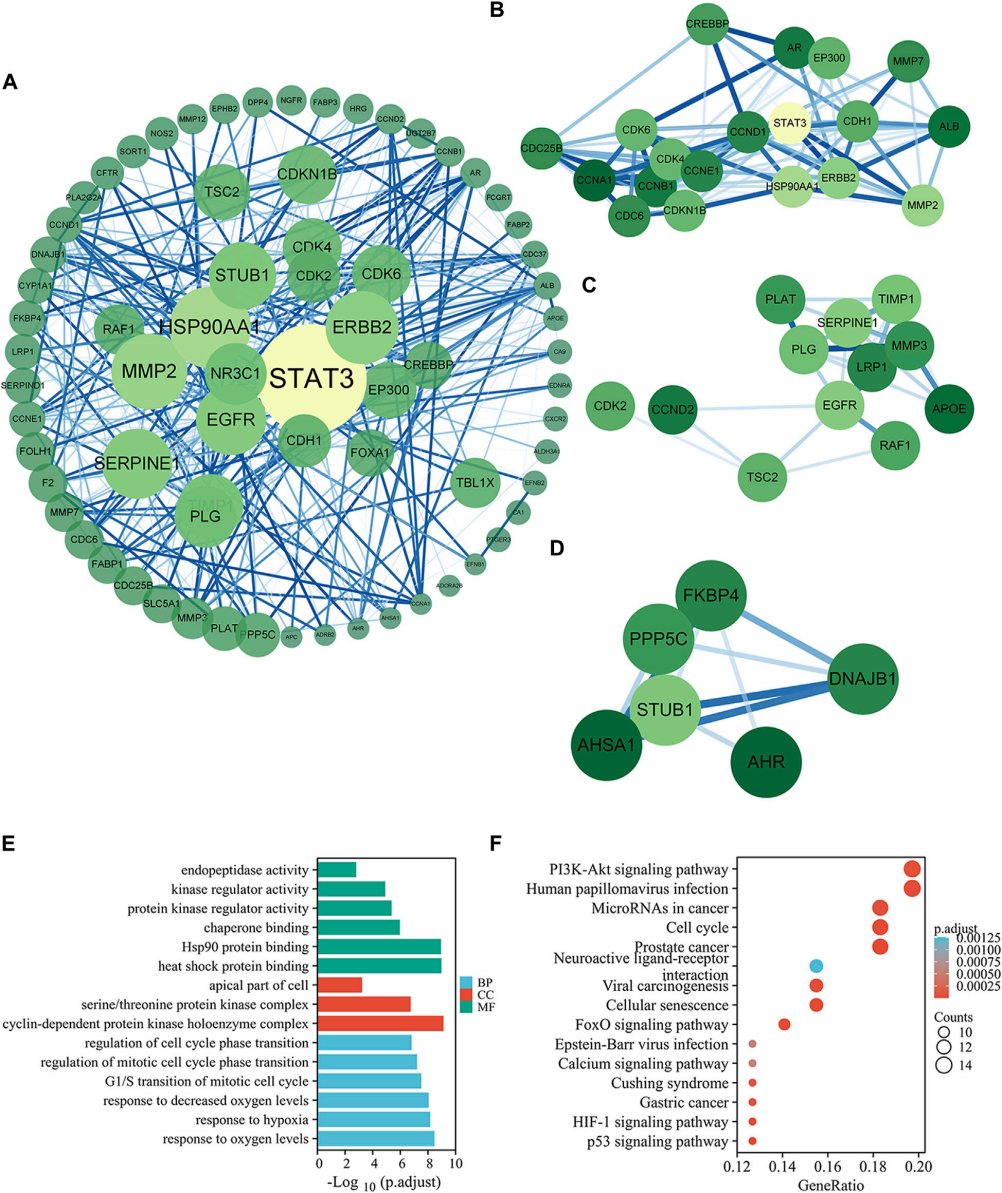
Potential targets and mechanisms of MPX halting the malignant transformation of PLGC. **(A)** PPI of the common targets related to PLGC interacting with MPX molecules. **(B–D)** Significant modules selected from PPI. **(E–F)** GO enrichment and KEGG pathway analysis for intersection targets of MPX and PLGC.

After prediction and expansion, 735 genes corresponding to 46 compounds were screened out as targets of MPX, and 654 genes were included as targets of PLGC. And 76 intersected genes of the above two was analyzed as potential targets. Then, the PPI network was created to systematically summarize the interactions of MPX targets associated with PLGC treatment (Figure 3A). Five targets were not analyzed for their absent interaction with other proteins. The network showed viable protein target nodes (*n* = 71) connected by edges (n = 467) with an average number of neighbors of 13.155 and average local clustering coefficient of 0.287. The top 10 genes by degree in network were STAT3, RSP90AA1, MMP2, ERBB2, EGFR, SERPINE1, STUB1, TIMP1, NR3C1, and PLG, which were considered crucial targets of MPX against PLGC. All related data refer to Supplementary Material S4.

Three significant modules were selected from the PPI network by MCODE. For module 1, the genes were enriched in cell cycle and PI3K-Akt signaling pathway (Figure 3B). Cluster 2 genes were enriched in cellular senescence and p53 signaling pathway (Figure 3C) and EGFR is the key upstream gene of the PI3K-AKT signaling pathway. Cluster 3 genes were enriched in protein processing in endoplasmic reticulum (Figure 3D). These data suggested that MPX likely acts on PLGC through above 5 signaling pathways.

To further investigate the multiple mechanisms of MPX at a systematic level, 76 common genes were analyzed with org. Hs.eg.db and clusterProfiler packages in R Studio. The top 6 terms in biological process, 6 terms in molecular function, and 3 terms in cellular component are presented in Figure 3E. GO enrichment analysis showed that the target genes were expressed in the apical part of cell, cyclin-dependent protein kinase holoenzyme complex, and serine/threonine protein kinase complex. At the molecular level, the target genes were involved in heat shock protein binding, protein kinase regulator activity, and kinase regulator activity. Moreover, biological processes were enriched in response to oxygen levels, decreased oxygen levels and hypoxia.

KEGG pathway annotation showed in Figure 3F. A total of 80.3% (61/76) potential target genes were enriched and involved in 66 pathways associated with the immune system, cancer, and inflammation. As the pathway with the highest enrichment score, PI3K-AKT signaling pathway was focused in the researches with its importance for cell growth, differentiation, metabolism, survival, and apoptosis.

### MPX Decreases the Neoplastic Incidence in PLGC Rats

**FIGURE 4:**
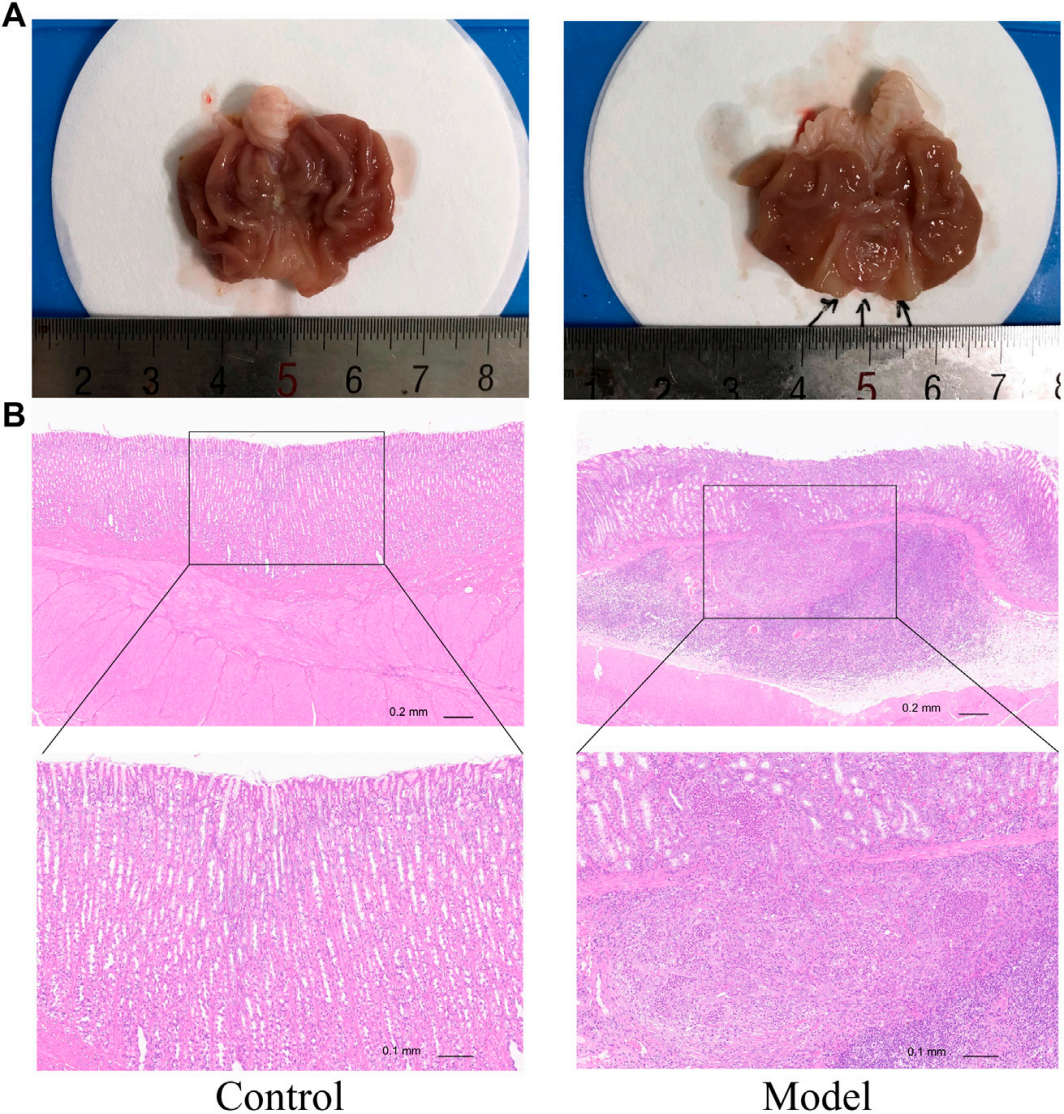
MNNG multiple factors-induced gastric mucosa dysplasia. **(A)** Gross evaluation of the gastric mucosa. **(B)** Microscopic appearance of the gastric epithelium stained by hematoxylin and eosin staining (magnification ×40, ×100).

We used above verified PLGC rats to further explore the anti-neoplasm formation efficacy of MPX. At the 32nd week, we tested the pathomorphology of gastric epithelium in control and model group rats on macroscopic and microscopic levels. As shown in Figure 4, gastric mucosa from normal control rats exhibited as bright red and shiny, thick and full. Under light microscope, the gland and cells appeared intact arrangement and morphology. By contrast, gastric mucosa from MNNG multiple factor-induced model rats exhibited as dull red, poor lustrousness, and rough-surfaced. Light microscope revealed distorted, crowded glands in which cellular atypia characterized by enlarged and hyperchromatic nuclei, and increased nuclear-cytoplasmic ratio and loss of polarity.

**FIGURE 5:**
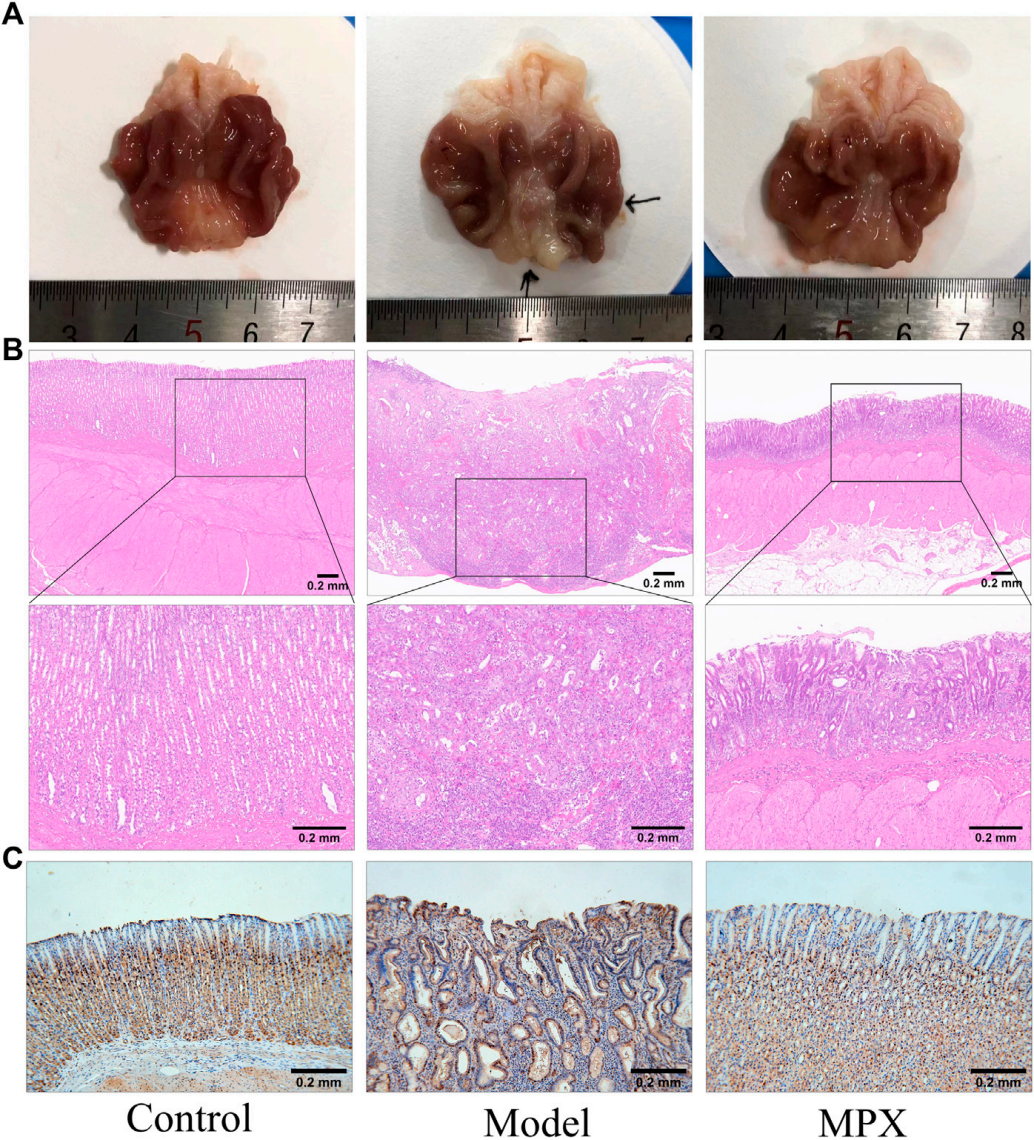
The level of gastric mucosal lesions and cell proliferation. **(A)** Gross evaluation of the gastric mucosa. **(B)** Microscopic appearance of the gastric epithelium stained by hematoxylin and eosin staining (magnification ×40, ×100). **(C)** Immunohistochemical analysis of gastric mucosa sections for PCNA.

After 8 weeks of medication, the oral administration of MPX decoction could reduce the neoplasm incidence from 71.43% (10/14) to 35.71% (5/14). Compared with the model group, the number of PLGC rats in the MPX group that progressing to gastric cancer was reduced by 50%. Although the *p*-value of this examination was 0.0656, we still considered this as evidence of anti-neoplasm effect of MPX. The pathology changes in different groups also showed that the MPX decoction had a significant effect of anti-neoplasm formation. There were obviously vegetations on the surface of gastric mucosa of the model group rat (Figure 5A). In addition, the pathology test revealed that the gastric mucosa in model group rats showed more variability in cell size, and the nucleus appears both larger and darker than normal cells. However, the morphology change of cells in MPX group were more regular compared with model group (Figure 5B). These findings indicated that MPX effectively halted the further deterioration of gastric mucosal histopathology in PLGC rats and reduced the incidence of gastric cancer.

Furthermore, we performed immunohistochemistry staining of PCNA in gastric tissues to assess the cell proliferation. Figure 5C demonstrated that PCNA was positively stained in cellular nucleus of normal gastric glands, particularly in the neck and deep layer areas, which are usually considered as the sites of progenitor cells localized. While, PCNA was significantly higher in model group and extended to whole glands. Obviously, MPX group showed moderate staining in the neck and deep layer areas. It is suggested that MPX effectively inhibits the malignant proliferation of gastric epithelial cells.

### MPX Alleviates the Systemic Inflammation in PLGC Rats

**FIGURE 6:**
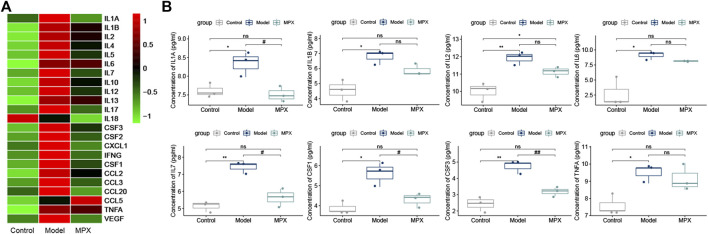
MPX alleviates the serum inflammatory cytokines levels in PLGC model rats. **(A)** Heat map of inflammatory cytokines expression levels in different groups; the raw data was normalized into Z-score, and the mapping has been colored according to their Z-score. **(B)** Histogram of serum inflammatory cytokines expression levels with significant differences. Compared with control group, *p < 0.05, **p < 0.01. Compared with model group, #*p* < 0.05, ##*p* < 0.01.

Since the chronic inflammation is highly associated with gastric precancerous lesions, we assessed the serum inflammation level in different groups with Luminex chip. Total of 23 key inflammatory cytokines were tested in the rat serum (Supplementary Material S5). As shown in Figure 6A, 21 cytokines were increased in the PLGC model group compared with control group, and among them, 12 cytokines were downregulated after MPX decoction intervention. Then, we performed further statistical analysis for the inflammatory cytokines. The histogram was used to display the cytokines with significant differences. Figure 6B showed that the proteins level of IL-1α, IL-1β, IL-2, IL-6, IL-7, CSF-1, CSF-3, and TNF-α were increased in PLGC model rats. After MPX decoction treatment, the levels of IL-1α, IL-7, CSF-1, and CSF-3 were recovered (*p*<0.05).

### MPX Downregulates the EGFR-PI3K-Akt Related EMT Pathway in PLGC Rats

**FIGURE 7:**
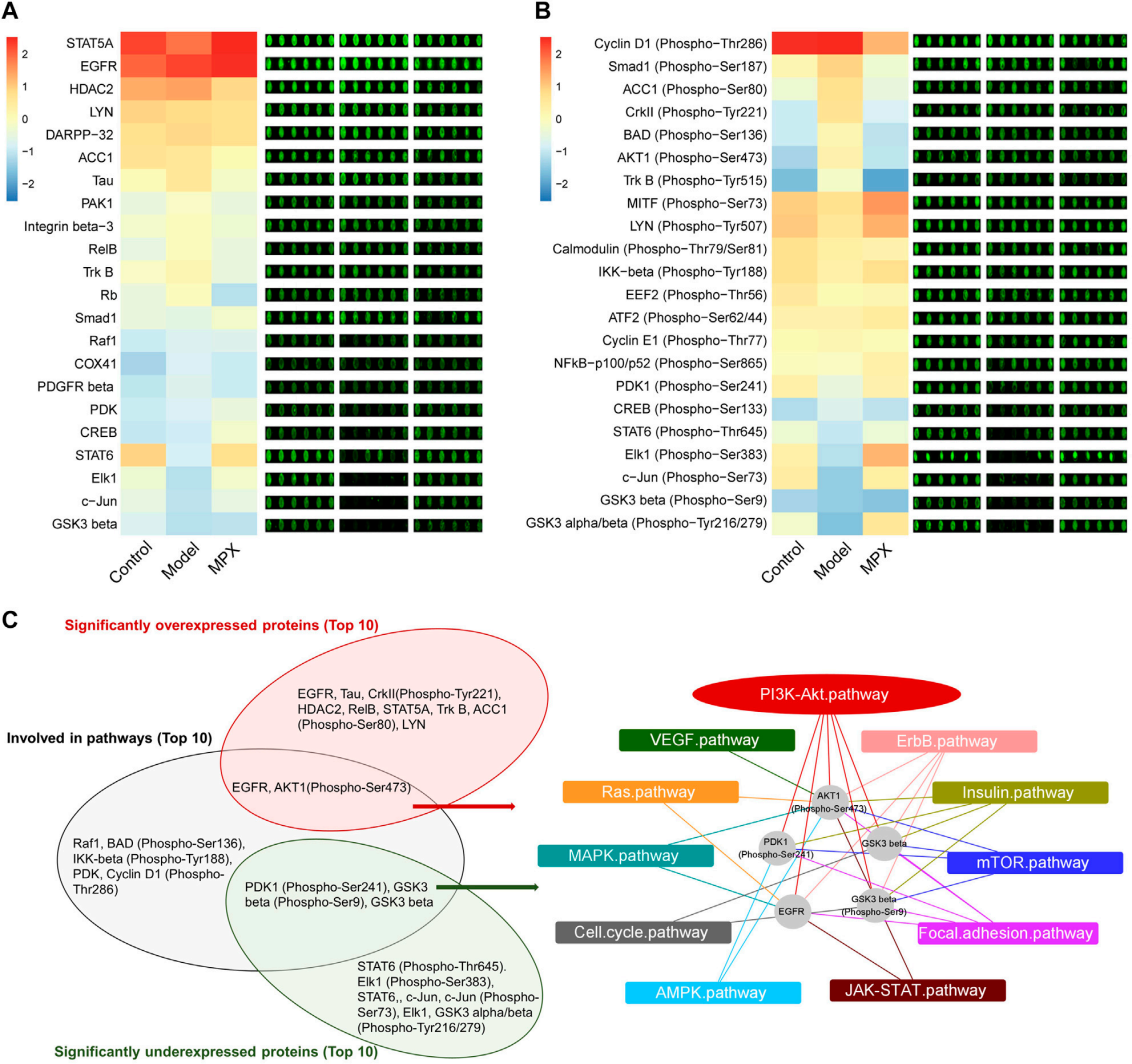
MPX regulates the protein expression and protein phosphorylation levels of tumor and immune-related signaling pathways in gastric mucosa. **(A)** The heatmap and fluorescence imaging of protein expression levels. **(B)** The heatmap and fluorescence imaging of protein phosphorylation levels. **(C)** Cluster analysis of the proteins with significant difference and summarization of core signaling pathways affected.

**FIGURE 8:**
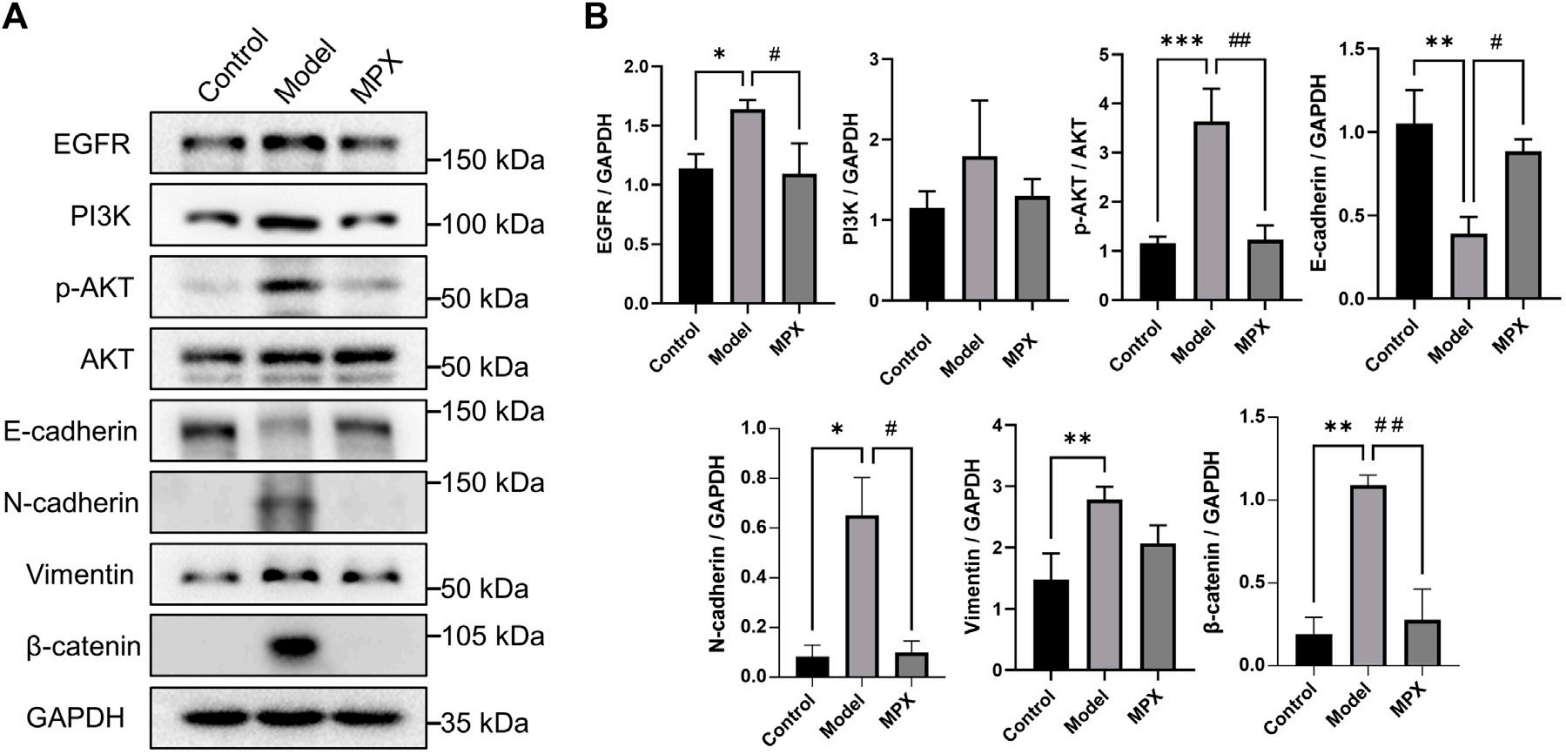
MPX downregulates the EGFR-PI3K-Akt related EMT pathway in PLGC model Rats. **(A–B)** Protein expression levels of EGFR, PI3K, p-AKT, AKT, N-cadherin, E-cadherin, Vimentin and β-catenin in gastric mucosa detected by western blot. Compared with control group, *p < 0.05, **p < 0.01, ***p < 0.001; Compared with model group, #*p* < 0.05, ##*p* < 0.01.

In order to explore the mechanism of MPX in the treatment of PLGC, we performed protein microarray detection on gastric mucosa tissues of the normal group, model group and MPX group, and analyzed 16 key tumor and immune-related signaling pathways (Supplementary Material S6). Figures 7A,B showed the heatmap of protein expression levels from 16 key tumor and immune-related signaling pathways and the fluorescence imaging of corresponding proteins expression. We screened the top 10 upregulated and downregulated proteins as well as the top 10 proteins that regulated the most signaling pathways. Then, we got the intersection between them as shown in Figure 7C. Cluster analysis was used to analyze the core differential proteins and signaling pathways. The results indicated that the EGFR-PI3K-Akt pathway and EMT pathway were highly associated with the anti-PLGC effect of MPX. Finally, western blot was used to further validate the EGFR-PI3K-Akt and EMT associated proteins involved in the treatment effects of MPX on PLGC. As shown in Figure 8, compared with control group, the protein levels of EGFR, p-AKT/AKT, β-catenin and N-cadherin were significantly increased and the expression level of E-cadherin was decreased in model group. MPX administration suppressed the changes in these indexes (*p* < 0.05).

## Discussion

Transformation from normal mucosa to neoplastic epithelium is a long-term process and several signaling pathways have been identified as the pivotal promoter in cancer formation (Hausman, 2019). The arising of precancerous lesions is the pre-step from cancer. Unlike many therapies to treat GC in guidelines, the therapy towards precancerous lesions is limited, especially lack of effective drug (Kapadia, 2003; Sung, 2016). Hence, how to manage patients with PLGC and find new drugs to block the progress of neoplastic cascade remains a challenge for gastroenterologists and researchers. TCM formula as an alternative method, has been suggested to treat patients with PLGC and has received good feedback in several clinical center. In this study, we integrated serum pharmacochemistry, bioinformatics, network pharmacology and experimental verification to explore the multi-target and multi-pathway regulating effects of MPX in the treatment of PLGC. We unveiled that the MPX could halt the progress of precancerous lesions to GC through inhibiting systemic inflammation and EGFR-PI3K-AKT related EMT pathway in local gastric mucosa.

To validate the efficacy and investigate the pharmacology of MPX against PLGC, the main active components identification is the key step. Mass spectrum, as a powerful instrument to identity different size of molecules, is widely used in identification of active principle in drug-contain serum (Yunxiang et al., 2018; Qin et al., 2021). In the study, we identified the characters of MPX-contained serum by Mass Spectrum. A total of 46 compounds in MPX-contained serum were obtained. Then, we analyzed the targets of these compounds using network pharmacology, and their intersection with precancerous lesions targets was obtained to reveal the mechanism of MPX in the treatment of PLGC. These results showed the multi-target and multi-pathway treatment effects of MPX against PLGC via PI3K-AKT signaling pathway, cell cycle, cellular senescence, p53 signaling pathway and protein processing in endoplasmic reticulum. However, it is still needed to further clarify the specific regulatory effects on these signaling pathways.

The current GC models (Hayakawa et al., 2013) can be divided into three types: chemical carcinogenesis model, *H. pylori* infection model and genetically engineered model. Among them, MNNG-induced GC model is the most common chemical carcinogenesis model and more appropriate to explore GC cascade mechanism. According to our previous researches, we applied the modified MNNG-induced GC model, which established through oral administration of MNNG, sodium salicylate, irregular fasting, and ranitidine, and such model rats exhibit the phenotypes of gastric mucosa pathology including atrophy, metaplasia, dysplasia, and adenocarcinoma (Chu et al., 2021; Wang et al., 2021). Then, we assessed the anti-precancerous lesions efficacy of MPX on a multi-factor induced PLGC model rats. Our results revealed that MPX could block the PLGC progress.

Considering the multi-target and multi-pathway treatment effects of TCM formula, we explored the underlying mechanisms of MPX against precancerous lesions by using serum cytokines chips and gastric mucosa protein chips. Unlike the local inflammatory response, which has been described thoroughly, the systemic inflammation rarely attracts researchers’ attention until recent years. However, it is still highly associated with cancer formation (Shinko et al., 2017; Tuomisto et al., 2019). MPX significantly decreased the level of serum inflammatory cytokines, indicated that MPX maybe a potential drug for inhibiting cancer-related systemic inflammation. But, the mechanism of MPX suppressed the serum inflammatory cytokines is not clear. Next, we used protein chips to explore the target signaling pathways of MPX decoction. Among all the vital targets in the progress of cancer formation, EGFR-PI3K-Akt signaling pathway and EMT signal were the two most relevant pathways after MPX treatment.

Abnormal amplification of receptor tyrosine kinases (RTKs) genes represented by EGFR is one of the typical molecular features in intestinal-type GC induced by *H. pylori* infection (Chichirau et al., 2019). The activation of EGFR and its downstream PI3K-AKT phosphorylation participated in the inflammatory process of gastric epithelial mucosa, and induced cellular consequences with carcinogenic potential (Polk and Peek, 2010; Ito et al., 2020). Now, Targeting EGFR is a therapeutic strategy in development in gastric cancer (Joshi and Badgwell, 2021). Our results showed the significant inhibitory effects of MPX on EGFR and PI3K-AKT pathways. PI3K-Akt signaling pathway is frequently altered in many human cancers. Many investigators have described that gastric cancer mediated by PI3K-AKT signaling pathway, which promotes the accumulation of β-catenin and the progression of gastric cancer (Vara et al., 2004; Polk and Peek, 2010). The activation of β-catenin has been reported as an early initiating event in gastric carcinogenesis and its accumulation in cytoplasm is related to EMT occurrence, which also regulated by PI3K-AKT pathway (Kolligs et al., 2002; Pearlman et al., 2017). EMT is a pathological phenomenon that often occurs in the cancer formation and metastasis. The existence of anchored protein and adhesive protein are necessary for cell to maintenance a normal phenotype, and the absence of such proteins could promote cell precancerous lesions and enhance cell migration and invasion properties (Gonzalez and Medici, 2014; Saitoh, 2018). In our study, MPX recovered the E-cadherin protein level and suppressed the expression levels of N-cadherin and β-catenin.

In summary, this study demonstrated that MPX is a potential drug for the treatment of PLGC. MPX could halt the progress of PLGC through alleviating the systemic inflammation and inhibiting EGFR-PI3K-AKT related EMT pathway in local gastric mucosa. These results provide the basis for clinical application of MPX in GC prevention.

## Data Availability

The datasets presented in this study can be found in online repositories. The names of the repository/repositories and accession number(s) can be found in the article/Supplementary Material. In addition, publicly available data sets were analyzed in this study. These data can be found here: TCGA and the GEO database.
