# Supplementary figures and images for: Manpixiao Decoction Halted the Malignant Transformation of Precancerous Lesions of Gastric Cancer: From Network Prediction to In-Vivo Verification
Source: Front Pharmacol. 2022 Aug 5;13:927731. doi: 10.3389/fphar.2022.927731 (PMC9389883; doi:10.3389/fphar.2022.927731)

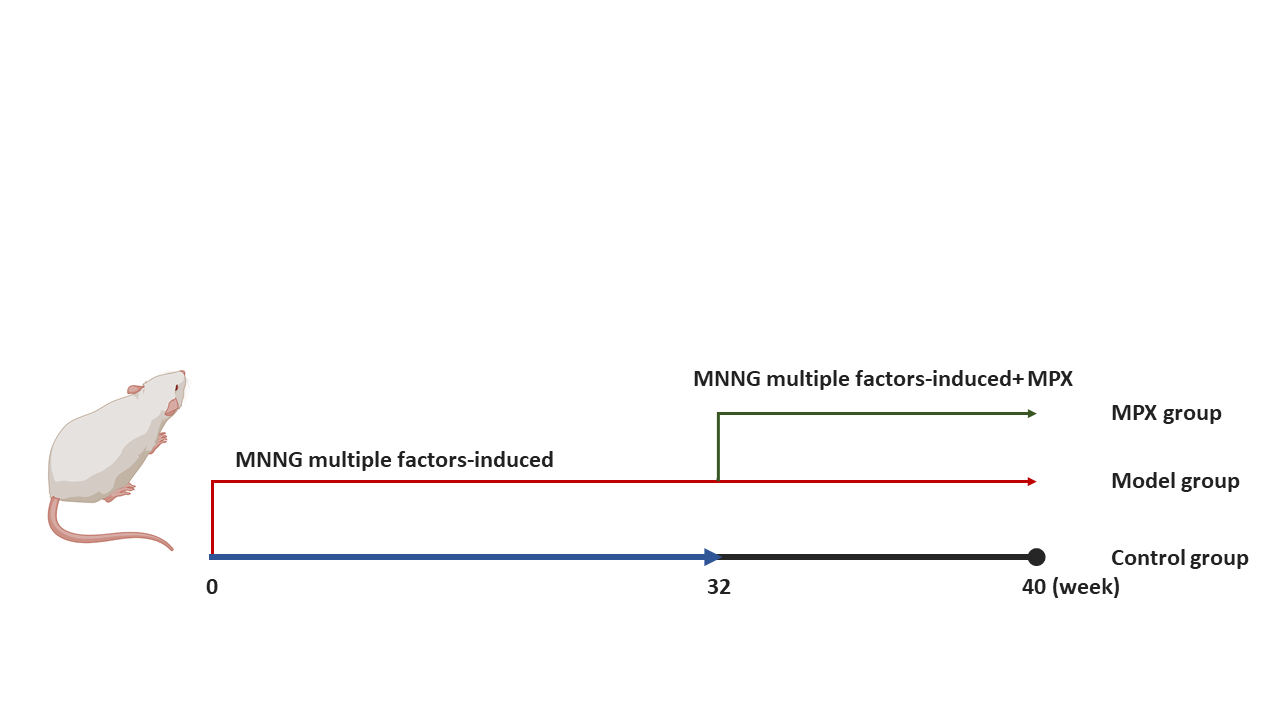

Supplement: Supplementary file 3 [file Image1.TIF]
